# Near-Infrared Laser Adjuvant for Influenza Vaccine

**DOI:** 10.1371/journal.pone.0082899

**Published:** 2013-12-11

**Authors:** Satoshi Kashiwagi, Jianping Yuan, Benjamin Forbes, Mathew L. Hibert, Eugene L. Q. Lee, Laura Whicher, Calum Goudie, Yuan Yang, Tao Chen, Beth Edelblute, Brian Collette, Laurel Edington, James Trussler, Jean Nezivar, Pierre Leblanc, Roderick Bronson, Kosuke Tsukada, Makoto Suematsu, Jeffrey Dover, Timothy Brauns, Jeffrey Gelfand, Mark C. Poznansky

**Affiliations:** 1 Vaccine and Immunotherapy Center, Division of Infectious Diseases, Department of Medicine, Massachusetts General Hospital, Charlestown, Massachusetts, United States of America; 2 Department of Pathology, Harvard Medical School, Boston, Massachusetts, United States of America; 3 Department of Applied Physics and Physico-Informatics, Faculty of Science and Technology, Keio Universtiy, Kohoku-ku, Yokohama-city, Kanagawa, Japan; 4 Department of Biochemistry, School of Medicine, Keio University, Shinjuku-ku, Tokyo, Japan; 5 SkinCare Physicians of Chestnut Hill, Chestnut Hill, Massachusetss, United States of America; Centers for Disease Control and Prevention, United States of America

## Abstract

Safe and effective immunologic adjuvants are often essential for vaccines. However, the choice of adjuvant for licensed vaccines is limited, especially for those that are administered intradermally. We show that non-tissue damaging, near-infrared (NIR) laser light given in short exposures to small areas of skin, without the use of additional chemical or biological agents, significantly increases immune responses to intradermal influenza vaccination without augmenting IgE. The NIR laser-adjuvanted vaccine confers increased protection in a murine influenza lethal challenge model as compared to unadjuvanted vaccine. We show that NIR laser treatment induces the expression of specific chemokines in the skin resulting in recruitment and activation of dendritic cells and is safe to use in both mice and humans. The NIR laser adjuvant technology provides a novel, safe, low-cost, simple-to-use, potentially broadly applicable and clinically feasible approach to enhancing vaccine efficacy as an alternative to chemical and biological adjuvants.

## Introduction

Safe and potent immunologic adjuvants are a key element of current vaccine design [1], [2]. While vaccination is considered effective as the primary strategy for the control of influenza infection [3]–[5], current influenza vaccines without adjuvant are efficacious in approximately 60% of patients [4], [6]. Importantly, efficacy is reduced in elderly and neonatal populations, where influenza-related complications and death is much higher than in other age groups [4], [7], [8]. Use of immunologic adjuvants in conjunction with influenza vaccines results in the increased generation of protective immunity, especially against emerging viruses with novel hemagglutinin (HA) sequences including H5N1 viruses [1], [3], [5], [9]–[13]. Unfortunately, while many development-stage adjuvanted vaccine formulations enhance vaccine efficacy, they also demonstrate significant side effects [14], [15]. Few adjuvanted vaccines are safe enough to merit approval by regulatory agencies [3], [10]. Tellingly, there is a paucity of effective adjuvants for influenza vaccine; the recent pandemic H1N1 influenza vaccine went through to production and implementation without an adjuvant [1], [5], [16], [17]. In light of these considerations, the development of new, safe and effective adjuvants is important for current and future vaccination programs.

In addition to new adjuvants, a variety of new vaccine delivery methodologies have been developed seeking to further optimize vaccine efficacy. This includes intradermal (i.d.) delivery of vaccine antigens which is proposed to induce superior protective immune responses in comparison to conventional intramuscular or subcutaneous delivery, as the dermis and epidermis are enriched with antigen-presenting cells (APCs) [18]–[20]. Work over the past three decades consistently report that i.d. delivery of reduced quantities of vaccine antigen can induce equivalent immune responses for vaccines including influenza, hepatitis B and rabies [18], [19]. Accordingly, i.d. delivered influenza vaccines, including Intanza® and IDflu® are now employed in more than 40 countries [21] and Fluzone Intradermal® was approved by the U.S. Food and Drug Administration (FDA) in 2011 [22]. However, the efficacy of the present form of i.d. influenza vaccine is comparable to the conventional vaccine delivered via the intramuscular route [19], [23]. Use of immunologic adjuvants could further increase the efficacy and dose-sparing potential of i.d. delivery. These potential benefits remain unrealized as the candidate adjuvants or adjuvants used in licensed vaccines are too reactogenic locally when delivered intradermally [19], [24]. Consequently, Intanza® or Fluzone Intradermal® do not contain adjuvant. Development of novel adjuvants designed for i.d. vaccines would therefore constitute a significant advance.

Previous work using visible range laser light illumination of the skin report enhanced immune responses to vaccination in humans and mice [24]–[26] and could be used as an immunologic adjuvant for i.d. vaccination. However, these lasers require co-administration of chemical adjuvant to achieve an effective immunological response [24]. In addition, laser light in the green or yellow spectrums is absorbed by melanin, resulting in highly variable light absorption across different skin phototypes, limiting the clinical utility of visible spectrum lasers [27]. Here we report that a continuous wave (CW), near-infrared (NIR) laser represents a new class of adjuvant that elicits a robust immune response without the use of other adjuvant agents independent of skin-phototype. In concert with i.d. vaccination, NIR laser adjuvants offer a feasible alternative to chemical adjuvants.

## Materials and Methods

### Animals

Seven-week-old female C57BL/6J mice were purchased from Jackson Laboratory. CD11c-eYFP mice were donated by Dr. Nussenzweig at Rockefeller University. All measurements were performed in a blinded manner, (to control or experimental groups).

### Laser illumination

We used a neodymium-doped yttrium orthvanadate (Nd:YVO_4_) laser (RMI Laser, Lafayette, CO). The 1064 nm laser can be set to emit either continuous wave (CW) output or nanosecond-duration pulses (PW) at a periodicity of 10 kHz, while at 532 nm the output is only PW. Average output powers were determined using a power meter for each illumination (Thorlabs). The beam profile for all exposures was flat, with a less than 50% variation in beam intensity from center to edge. The laser diameter on the skin was measured approximately 5 mm (0.2 cm^2^). Mice were depilated using a hair remover (Nair, Church & Dwight). The following day, the shaved skin of anesthetized mice was illuminated with the laser on 4 spots for ovalbumin (OVA) and 1 spot for influenza studies. The skin temperature was measured during the procedure using an infrared thermal imager (FLIR Systems).

### Skin damage study

For visual inspection, we observed for any signs of skin damage including blistering, bruising, crusting, edema, redness or swelling during and at 0, 1, 2, and 4 days after laser illumination. For skin histology, mice were heart-perfused with 4% paraformaldehyde before, or at 3, 6, 24, 48, and 96 hours after laser illumination. Five µm-thick paraffin sections were H & E stained and examined for microscopic tissue damage, and polymorphonuclear infiltration were quantitated on the slides in 5 randomized fields using Image J freeware (NIH).

### OVA immunization

Mice were injected intradermally (i.d.) using a 28 G insulin grade syringe (Kendal) with chromatographically purified OVA (10 µg in 10 µL saline per spot, 4 spots, Worthington), which was found to contain less than 1.75 EU/mg of endotoxin using the Limulus amebocyte lysate QCL-1000 (Cambrex). I.d. delivered alum- (Imject®, Thermo-Fisher) adjuvanted OVA, prepared per manufacturer's instructions, served as positive control. A dose of 5 µL of Imject® (200 µg aluminum hydroxide plus 200 µg magnesium hydroxide) was used per spot. Blood samples were drawn at 3, 6 and 12 weeks post-vaccination via retro-orbital bleeding.

### Influenza immunization

Mice were injected i.d. with whole inactivated influenza virus A/PR/8/34 (H1N1) (1 µg in 10 µL saline, 1 spot, Charles River). Alum-adjuvanted vaccine served as a positive control. Blood samples were taken 28 days after immunization and 4 days post-challenge with an intranasal application of live influenza virus 4 weeks after vaccination as previously performed in the context of i.d. influenza vaccination [28]–[31].

### ELISAs for quantitating anti-OVA and anti-influenza antibodies

Immulon™ 2 HB Flat Bottom Plates (Thermo-Fisher) were coated overnight with 1 µg of OVA at a concentration of 5 µg/mL or 0.2 µg of inactivated influenza virus at 1 µg/mL. Serially diluted mouse serum samples were added to the wells and incubated for 1 hour after the plates were blocked. Bound immunoglobulins were detected with the appropriate horseradish peroxidase-conjugated secondary antibody (goat antibody to mouse IgG [1:10,000, Sigma-Aldrich]; rat antibody to mouse IgG1 [1:4,000, SouthernBiotech]; rat antibody to mouse IgG2b [1:500, SouthernBiotech]; goat antibody to mouse IgG2c [1:4,000, SouthernBiotech]; goat antibody to mouse IgA [1:1,000, Sigma-Aldrich]; rat antibody to mouse IgE [1:1,000, SouthernBiotech]). In the case of IgE, the wells were further treated with ELAST ELISA Amplification System (Perkin Elmer) to improve sensitivity of the assay. At the end of the incubation, TMB substrate (1-Step Ultra TMB, Thermo-Fisher) was added and the reaction was stopped with 2 N sulfuric acid. The reproducibility of the assay was ascertained by applying mouse anti-ovalbumin IgG (Sigma-Aldrich) or a hyperimmune mouse serum to influenza to each plate. We measured the absorption at 450 nm using an ELISA reader (TECAN Sunrise™ plate reader, TECAN). For antibody titers to OVA and IgE antibody to influenza, a statistically defined endpoint antibody titer was determined with a confidence level of 99% as previously described [32]. For antibody titers to influenza except IgE isotype, a titer was designated as a serum dilution corresponding to an inflection point.

### Hemagglutination inhibition (HAI) titration

Mouse sera were analyzed for HAI titers by Charles River Avian Vaccine Services as described previously [33], [34].

### Influenza virus challenge study

Mice were anesthetized and challenged intra-nasally with live influenza A/PR/8/34 at a dose of 1.5×10^6^ 50% egg infectious doses (EID_50_), which is equivalent to 3×10^3^ 50% mouse lethal dose (MLD_50_), in 30 µL saline 28 days after vaccination. Survival and body weight were monitored for 14 days post-challenge. Mice showing a hunched posture, ruffled fur, or greater than 20% body weight loss, or mice which were not eating or drinking, were considered to have reached the experimental end point and were euthanized [33], [34]. MLD_50_ titers were determined by inoculating groups of 10 mice intranasally with serial 10-fold dilutions of virus using the Reed-Muench formula as previously described [33], [34].

### Influenza virus titration in lung homogenate

Four days after the virus challenge, both sides of the lung were isolated and homogenized. The EID_50_ m/L values were determined by serial titration of the lung homogenate in eggs by Charles River Avian Vaccine Services as described previously [33], [34].

### Splenocyte stimulation and intracellular cytokine staining

Splenocytes were harvested 4 days after the virus challenge as previously performed in the context of i.d. influenza vaccination [28]–[31]. The 2×10^6^ splenocytes were re-suspended in 100 µl of media and incubated for 5 hours in the presence of an inhibitor of Golgi function (Golgi plug, BD Bioscience) and 1 µg/mL of influenza A MHC class I (NP_366–374_, ASNENMETM, Anaspec), or II peptides (NP_311–325_, QVYSLIRPNENPAHK). Multi-parameter surface staining for CD3ε, CD4, and CD8α (CD3ε: 145-2C11; CD4: RM4-5; CD8α: 53-6.7, BD) was performed, followed by fixation, permeabilization in Cytofix/Cytoperm™ (BD Bioscience) according to the manufacturer's instructions and intracellular staining for IFN-γ, IL-17A, IL-5 (IFN-γ: XMG1.2; IL-17A: TC11-18H10; IL-5: TRFK5, BD). Cell subpopulations, including influenza-specific IFN-γ, IL-17A, IL-5 producing CD3+CD4+T-helper cells and CD3+CD8+T-cytotoxic cells, were quantified as a percentage of total viable cells by flow cytometry using a BD 4 Laser LSR II (BD). Analysis was completed using FlowJo software (Tree Star).

### Quantitation of dendritic cell migration and function in vivo

Mice were injected with OVA labeled with Alexa Fluor 647 (OVA_647_, 10 µg in 10 µl saline per spot, 4 spots in total, Invitrogen) with or without laser illumination. 24 hours after vaccination, skin-draining lymph nodes (dLNs) were harvested, minced, and stained for the cell surface and maturation markers CD11c, CD86, CD80, CD40, and I-A^b^ (CD86: GL1; CD80: 16-10A1; I-A^b^: AF6-120.1; BD, CD40: 1C10; CD11c: N418, eBioscience). We then performed flow cytometry on CD11c-positive dendritic cells (DCs).

To examine temporal and spatial expression of CCL2 and CCL20, and to quantify DCs in laser-treated skin, we performed immunofluorescence and confocal microscopic analysis of laser-treated skin from CD11c-eYFP mice, in which DCs are intravitally labeled [35], [36]. Control animals received sham treatments in which they were anesthetized and shaved, but not treated with the laser. Mice were heart-perfused with 4% paraformaldehyde in PBS via the left ventricle before, or at 6 or 24 hours after 1 minute of CW 1064 nm laser treatment. The skin was then harvested and embedded in OCT compound (Tissue-Tek, Sakura). The primary antibodies used were rat anti-mouse CCL2 (1:50, R&D Systems) or rat anti-mouse CCL20 (1:10, R&D Systems). Goat anti-rat IgG (Dylight 549, 1:200, Jackson Immunoresearch) was the secondary antibody used. 10 µm sections were washed and incubated with the respective primary antibody overnight at 4°C followed by a wash and 1 hour incubation at room temperature with the secondary antibody. Appropriate negative controls were prepared by omission of the primary antibody. Following application of the secondary antibody, tissues were washed, counterstained with To-Pro-3 (1:5000, Invitrogen) and mounted. Digital images of immunofluorescence slides were obtained by means of confocal microscopy (Carl Zeiss LSM5 Pascal; Carl Zeiss, Inc.). YFP-positive DCs were manually counted in 5 randomized fields using Image J 1.43 freeware (NIH).

### Quantitative PCR analysis

Skin sections measuring 5×5 mm^2^ and including both the epidermis and dermis were excised 6 hours after laser illumination. Total RNA was extracted using the RNeasy Fibrous Tissue Mini Kit (Qiagen) and reverse-transcribed using the RT^2^ First Strand Kit (Qiagen). The samples were tested on an RT^2^ Profiler™ PCR Array System (Qiagen) on an Mx3005™ Multiplex Quantitative PCR System (Stratagene). The fold change in mRNA expression over sham-treated controls was normalized against housekeeping genes and calculated following the 2^–ΔΔCT^ method.

### Safety study of NIR laser exposure in humans

We performed an open-label, single-center, single-arm study in healthy adults. We used a clinically-approved Q-YAG 5 laser emitting light at 1064 nm (Palomar). The laser was altered to operate at an average power level below 2 W with an exposure area of 0.5 cm^2^, pulse duration of 3 nanoseconds, and pulse frequency of 10 Hz. We selected qualified subjects with either skin phototype V or VI [37]. We exposed each subject to a range of doses by increasing the average irradiance level of each exposure from 0.5 to 3.7 W/cm^2^ in stepwise increments of about 0.2–0.4 W/cm^2^ each for up to 120 seconds. While subjects were asked to immediately report any sensations during and after the exposure, the operator recorded any signs of visible skin damage. An individual test exposure was stopped if there were any indications of the subject's discomfort, pain, or distress, or if the investigator noted any signs of skin damage. At the end of the exposure, the operator acquired a digital photograph of the area and scored the skin sensations and damage. Subjects returned 2 days later for follow-up assessments of their skin conditions and an additional digital photograph of the area. Subjects came in after 2 weeks if the subject indicated any appearance of skin markings in the area of the laser exposure.

### Ethics Statement

All animal procedures were performed following the Public Health Service Policy on Humane Care of Laboratory Animals and approved by the Institutional Animal Care and Use Committee of Massachusetts General Hospital. The laser safety study in humans was performed in full conformity with the Declaration of Helsinki, the current International Conference for Harmonisation Good Clinical Practice (ICH-GCP) regulations and all applicable regulatory and ethical requirements. The study was approved by New England Institutional Review Board (NEIRB) and the registry number is #09-325. All subjects provided written informed consent before study-related procedures were performed.

### Statistical analysis

We used the Mann Whitney U-test for the comparison of numerical values between 2 groups, and the Kruskal-Wallis followed by the Dunn's test for comparisons of more than three groups for all statistical analyses unless otherwise specified. Data were pooled from at least two independent experiments.

## Results

### Delineation of non-tissue damaging dosages of NIR laser parameters

**Figure 1:**
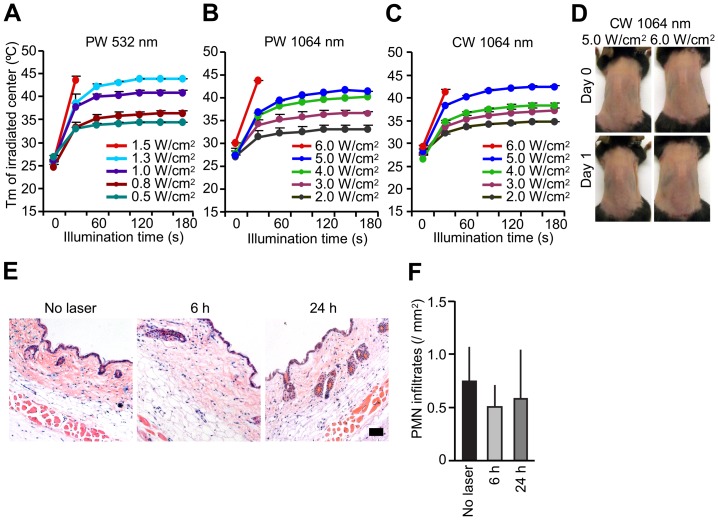
Effect of laser on skin tissue. A–C, Dose-temperature responses of the PW 532 nm laser and the PW and CW 1064 nm laser in mouse skin. *n* = 1–4 (4–16 exposures in total) for each group. PW, pulse wave; CW, continuous wave; Tm, maximal skin surface temperature. Error bars show means ± s.e.m. D, Images of the back of mice for visual inspection at 0 and 24 hours after the CW 1064 nm NIR laser treatment. Representative images for each group are presented. A–D, *n* = 1–4 (4–16 exposures in total) for each group. E, Microscopic assessment of skin damage and inflammatory infiltration after laser treatment. Representative time-course images of hematoxylin-eosin-stained skin tissue are presented. The bar indicates 50 µm. F, Quantification of polymorphonuclear leukocytes (PMN) after the NIR laser treatment. E–F, *n* = 3 for each group.

We first sought to establish the maximum non-tissue damaging dosages for the near-infrared (NIR) laser for both continuous wave (CW) and nanosecond-duration pulse wave (PW) mode as well as the previously described PW visible lasers [25], [26]. Mice received exposures at escalating irradiances (0.5 to 1.5 W/cm^2^ for PW 532 nm laser; 0.5 to 6.0 W/cm^2^ for CW or PW 1064 nm lasers) for durations up to 4 minutes. Skin damage was evaluated after illumination by visual inspection and histology. Maximum safe irradiances were considered to be those at which skin temperatures did not exceed 43°C and for which no visible or microscopic skin damage was apparent at any of the post-exposure evaluation times [27], [38]. We identified 1.0 W/cm^2^ as the maximum safe irradiance for the PW 532 nm laser (Figure 1A) and 5.0 W/cm^2^ for both the 1064 nm PW (Figure 1B) and CW lasers (Figure 1C). No visual damage such blistering, bruising, crusting, edema, redness or swelling damage was seen when the laser power was below the safe irradiance for each parameter for the PW 532 nm laser (Figure S1A) and 5.0 W/cm^2^ for both the 1064 nm PW (Figure S1A) and CW lasers (Figure 1D). On a histological examination, no tissue damage or inflammatory response at any given time point was detected by H & E staining (Figure 1E) with minimal polymorphonuclear cell-infiltration in the skin after the administration of 5.0 W/cm^2^ of CW 1064 nm laser (Figure 1F). Thus, we concluded that the dosages below 1.0 W/cm^2^ for the PW 532 nm and 5.0 W/cm^2^ for both the 1064 nm PW and CW lasers are non-tissue damaging and non-inflammatory.

### Safety and tolerability of the non-tissue damaging dosage of NIR laser in humans

**Figure 2:**
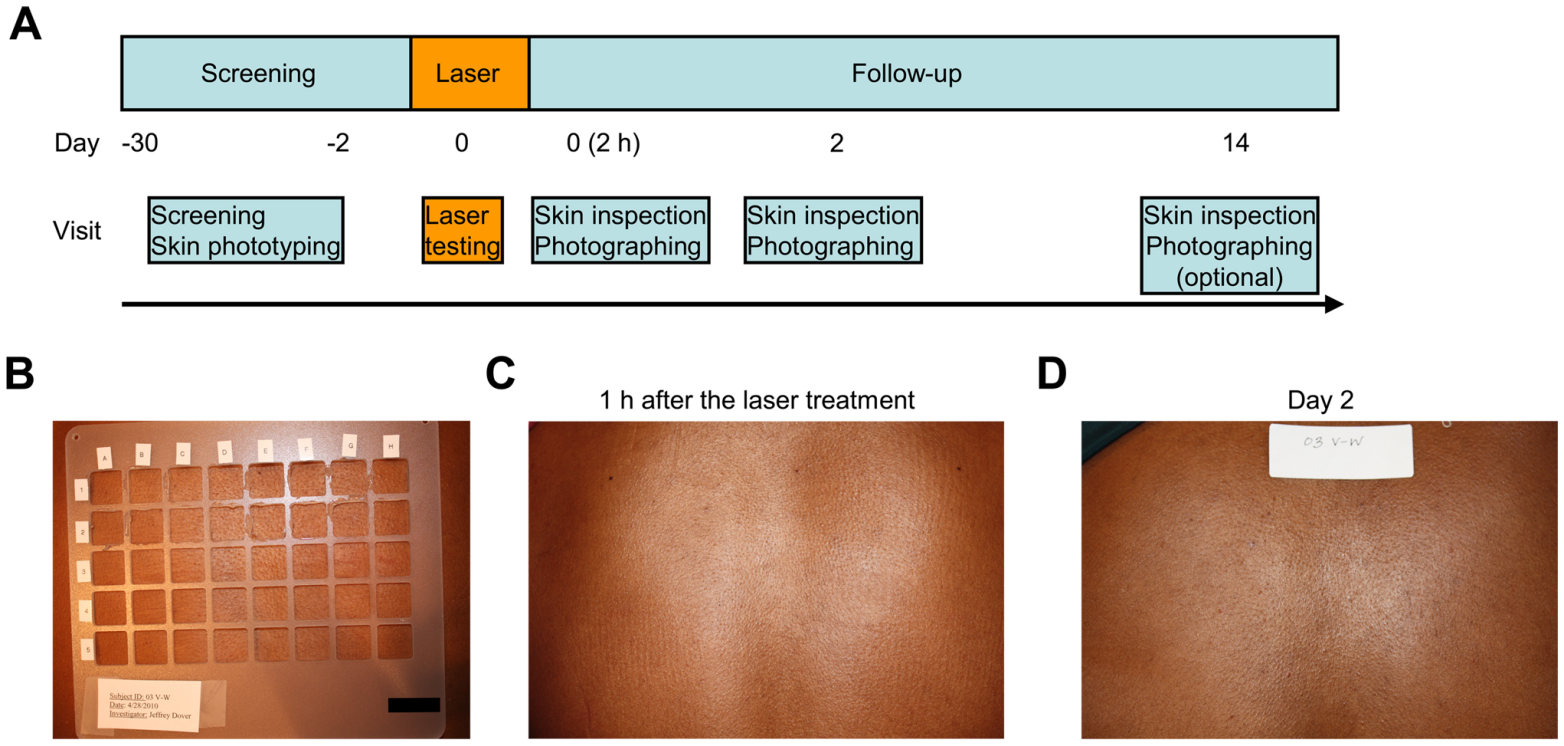
NIR laser safety study in humans. A, Schedule of laser treatment and follow-up skin appearance documentation. Five healthy adults aged 20 to 46 years old with either skin phototype V or VI were enrolled. B, A plastic grid was used to separate the laser exposure sites. An aqueous gel was applied in each section of the grid to enhance the dissipation of heat from the skin's surface. The bar indicates 1 inch. C and D, Representative images of the laser-exposed skin are shown at (C) 1 hour and (D) 2 days after completion of the treatment. No detectable skin damage on visual inspection was observed following laser exposure at any irradiance used.

In order to determine if the NIR laser dose used in mice is safe and tolerable in humans, we performed a clinical study (Figure 2A). Five subjects with skin phototypes V or VI were enrolled [37], and each subject received 16 exposures of a clinically-approved 1064 nm laser at escalating irradiances (0.5 to 3.7 W/cm^2^) for durations up to 2 minutes each (Table 1). All subjects tolerated the highest irradiance for 2 minutes (total dose 442 J/cm^2^) with no subject reporting severe skin sensations or distress (Table 2). Investigators noted no significant skin damage during any laser exposure (Figure 2B–D). Laser-induced skin damage is a function of skin heating, which depends upon the duration of exposure, wavelength and irradiance [38]. With the same wavelength and a similar order of irradiance and the duration of exposure, the heat generation in the animal and human studies is equivalent. Thus, we conclude that a NIR laser at equivalent irradiances and doses used in mice is safe and well tolerated in humans.

**Table 1:** List of enrolled subjects and tolerated doses.

Patient number	Age	Gender	Skin phototype	Tolerated irradiances (W/cm^2^)	Maximum tolerated fluences (J/cm^2^)	Premature termination of the laser exposure
1	24	F	V	0.5**–**3.7	441.6	No
2	21	F	V	0.5**–**3.7	441.6	No
3	46	F	VI	0.5**–**3.7	441.6	No
4	34	F	V	0.5**–**3.7	441.6	No
5	20	F	V	0.5**–**3.7	441.6	No

Study subjects were screened for inclusion and exclusion criteria detailed in Materials and Methods. Subjects were selected with either skin phototype V or VI because, at 1064 nm, levels of laser power and exposure time that proved to be non-painful and non-damaging in subjects with the darkest skin types would be predicted to be non-painful and non-damaging for all other skin types.

**Table 2:** Reported sensations and signs of skin damage on each NIR laser dose.

	Subjects					Number of sensations			Total events
Laser irradiances (W/cm^2^)	1	2	3	4	5	Mild	Moderate	Severe	
0.5						0	0	0	0
0.7						0	0	0	0
0.8						0	0	0	0
1.1						0	0	0	0
1.4					1	1	0	0	1
1.5					1,2	1	1	0	2
1.7						0	0	0	0
2.2			4			1	0	0	1
2.5	4		4			2	0	0	2
2.7			4	4	1	3	0	0	3
2.9	1,2		1,2	4	1,2	4	3	0	7
3.1	1,2		1	4		3	1	0	4
3.3	1			4	1,2	3	1	0	4
3.4	1,9	4,9	1			3	0	0	5
3.5	9	4,9	1	1	1,2	4	1	0	7
3.7	2,9	9	1,2	1	1,2,9	3	3	0	9

Consenting human subjects were exposed to a range of laser doses from 0.5 to 3.7 W/cm^2^ (16 doses) each up to 120 seconds. Sensations felt by subjects were classified as mild (warmth, tingling, itching, pinprick/needle sensations), moderate (hotness, dull pain), or severe (burning, sharp pain), and recorded. The operator also recorded any signs of skin damage.

1 Warmth

2 Hotness

3 Burning

4 Pinprick/needle sensations

5 Dull pain

6 Sharp pain

7 Tingling

8 Itching

9 Skin appearance change; No changes in skin appearance or damage were noted on all exposures, except transient skin darkening (transient hyperpigmentation) occurred in some subjects, which was due to changes in capillary blood flow in the treated area. These changes were not observed during follow-up examination after 2 hours.

10 Skin damage

### NIR laser adjuvant enhances antibody response to a model vaccine

**Figure 3:**
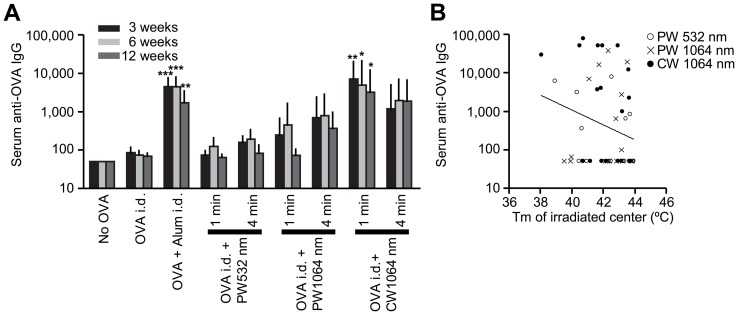
Effect of laser on the humoral immune response to a model vaccine. A, Serum ovalbumin- (OVA)-specific IgG titers 3, 6, and 12 weeks following vaccination with 40 µg OVA with or without laser illumination. Endpoint titer of OVA-specific serum IgG was determined by ELISA. Plates were coated with OVA. *n* = 33, 19, 15, 12 and 11, 5 and 6, 6 and 6 for no OVA, OVA i.d., OVA +Alum i.d., OVA i.d. + PW 532 nm 1 and 4 minutes, OVA i.d. + PW 1064 nm 1 and 4 minutes, OVA i.d. + CW 1064 nm 1 and 4 minutes, respectively. Error bars show means ± s.e.m. *P<0.05, **P<0.01 and ***P<0.001 as compared to OVA i.d. B, The relationships between anti-OVA antibody titers following 1–4 minutes PW 532 nm, PW 1064 nm and CW 1064 nm laser-treated groups at 6 weeks (logIgG) and maximal skin surface temperature (Tm) was not statistically significant; a Pearson's correlation coefficient *r* = −0.237 (*P* = 0.08), where log(IgG) = −0.193Tm+10.75 (linear regression, R^2^ = 0.056). *n* = 20, 17, 18, for OVA i.d. + PW 532 nm 1–4 minutes, OVA i.d. + PW 1064 nm 1–4 minutes, OVA i.d. + CW 1064 nm 1–4 minutes.

We next tested whether NIR laser treatment at safe irradiances and doses could enhance immune responses to vaccination in mice as compared to the licensed adjuvant, alum. First, mice received 1- or 4-minute exposures to CW or PW 1064 nm or PW 532 nm lasers on the back skin. Immediately thereafter, mice received an i.d. injection of OVA. Alum-adjuvanted OVA i.d. served as positive controls as i.d. injection of alum has been used to increase the efficacy of vaccines both in mice [39]–[42] and humans [43], [44]. Notably, the 1-minute CW 1064 nm laser treatment induced the highest antibody titer among all the tested parameters, which were significantly higher at all time points than both the non-adjuvanted controls (Figure 3A, 3 weeks: *P*<0.01; 6 weeks: *P*<0.05, 12 weeks: *P*<0.05). The anti-OVA specific IgG antibody titer in the previously explored 4-minute PW 532 nm laser-treated group was not significantly higher than those in non-adjuvanted controls at any time point (Figure 3A). There is no significant difference between CW 1064 nm laser-treated and alum-adjuvanted groups at any time point (Figure 3A). We did not find a relationship between maximal skin surface temperature during laser treatment and antibody titer (Figure 3B), suggesting that heat generation as a result of laser exposure does not play a significant role in enhancing immune responses. We selected the 1-minute CW 1064 nm laser exposure as the most effective and efficient immune adjuvant for subsequent experiments.

### NIR laser adjuvant induces functional changes in DCs

**Figure 4:**
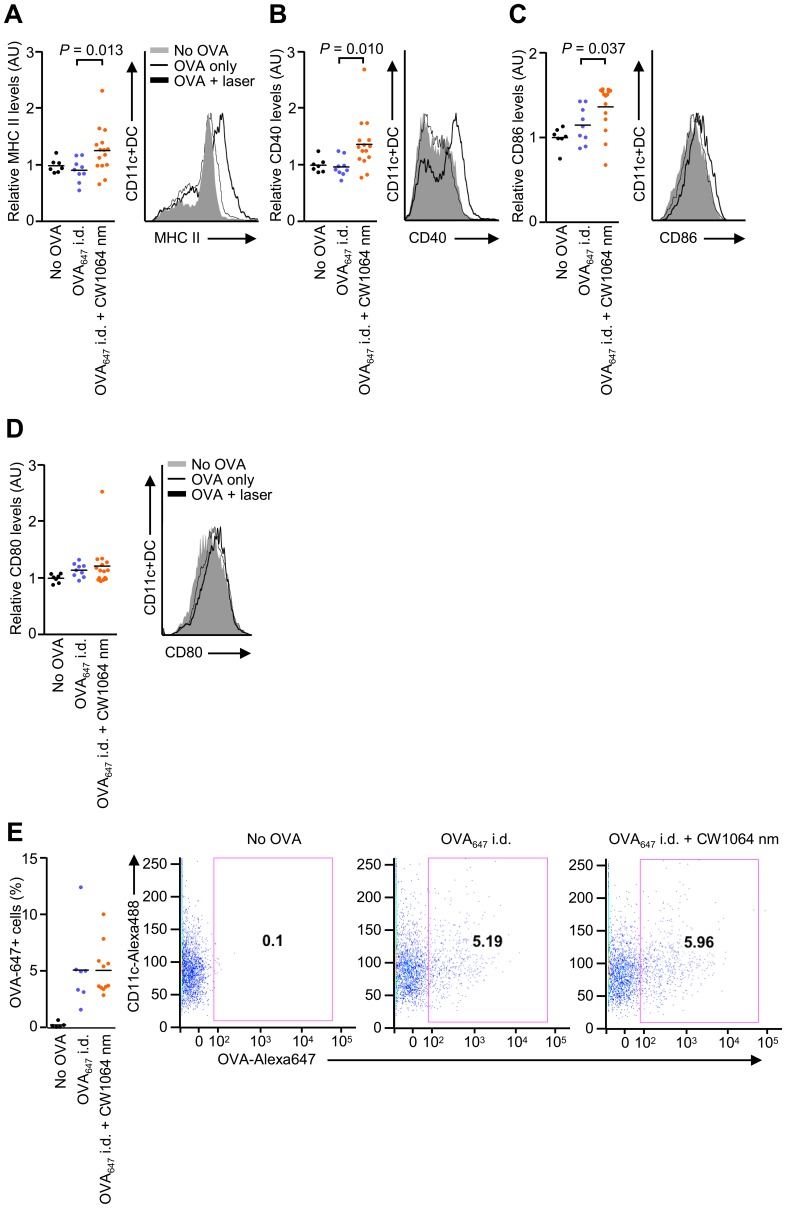
Effect of the near-infrared (NIR) laser adjuvant on the function of dendritic cells (DCs). A–E, Quantitation of DC activation markers (A) MHC class-II, (B) CD40, (C) CD86 and (D) CD80, and (E) the number of CD11c+OVA_647_+ DCs in skin-draining lymph nodes 24 hours after vaccination with 40 µg Alexa Fluor-647-labeled OVA (OVA_647_) with or without the 1-minute CW 1064 nm NIR laser treatment. Data are the ratio of median fluorescence intensity (MFI) of each marker normalized to no OVA controls. *n* = 7, 9, 15 for no OVA, OVA_647_ i.d., and OVA_647_ i.d. + CW 1064 nm, respectively; ANOVA with Bonferroni correction. AU, arbitorary units. A–E, Data are derived from at least three independent experiments.

**Figure 5:**
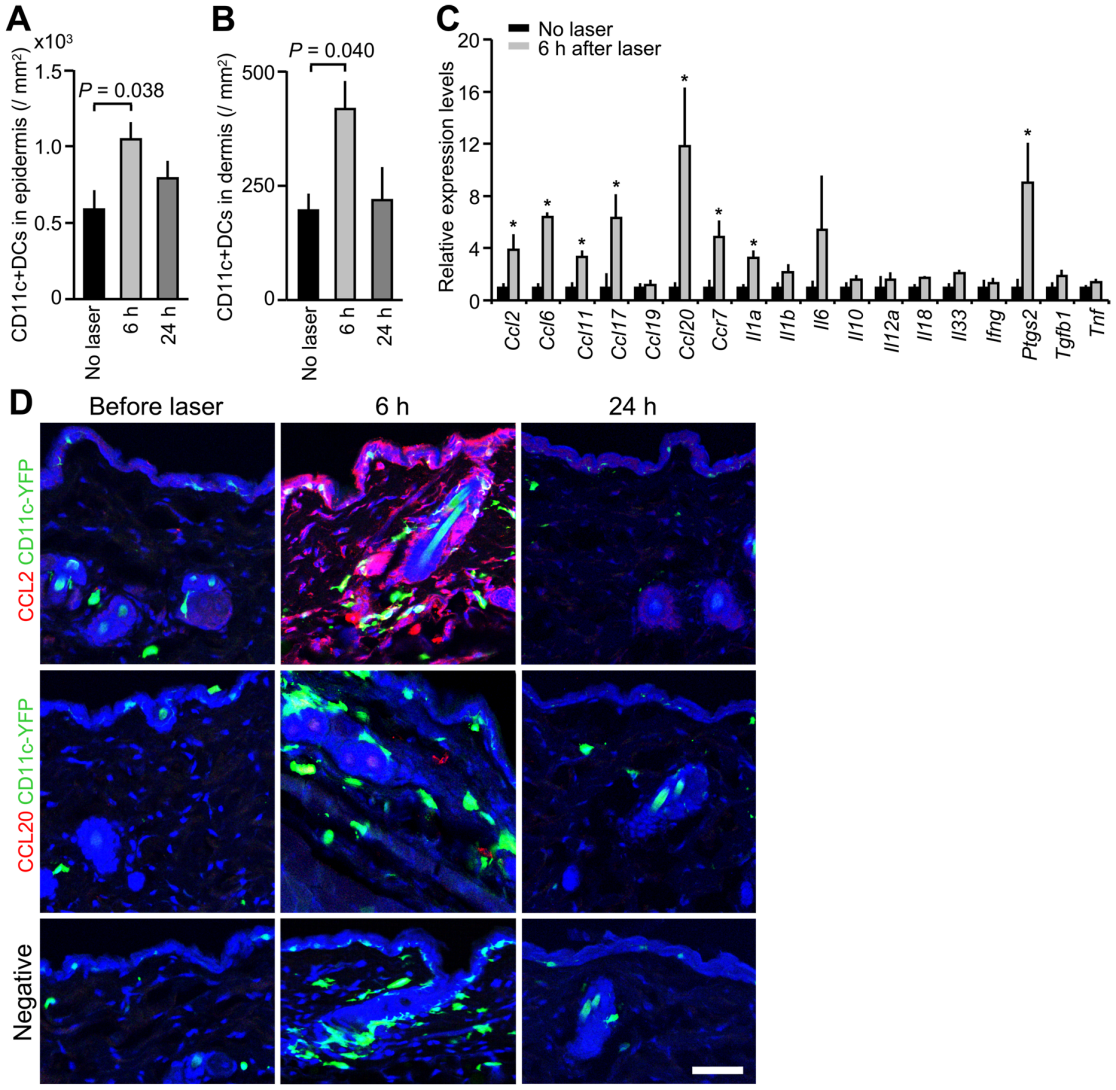
Effect of the near-infrared (NIR) laser adjuvant on the migration of dendritic cells (DCs). A and B, Quantification of CD11c+ DCs in skin before (*n* = 6) and at 6 (*n* = 8) and 24 hours (*n* = 4) after the NIR laser treatment. The number of DCs in the (A) epidermal and (B) dermal compartments; ANOVA with Bonferroni correction. A–B, Data are derived from at least three independent experiments. C, Relative gene expression of inflammatory cytokines and chemokines in the skin 6 hours after laser treatment (*n* = 4–5) and in the no laser control mice (*n* = 4) was quantified by qPCR. *P<0.05 as compared with control mice; Student's unpaired two-tailed *t* test. A–C, Error bars show means ± s.e.m. D, Confocal imaging of CCL2 expression (red, top row), CCL20 expression (red, middle row), CD11c+ DCs (green) and nuclear counterstaining (To-Pro-3 in blue) in skin before treatment and at 6 and 24 hours after the NIR laser treatment. Images are representative from three independent experiments. Scale bar, 50 µm.

Previous studies indicate that licensed and experimental immunologic adjuvants activate DC-mediated innate immune responses resulting in robust adaptive immune responses [3], [10], [45]–[47]. To investigate if the NIR impacts DC trafficking and function, we injected fluorescently labeled OVA i.d. into mice and assessed both OVA-positive DCs in skin-dLNs and their activation status. The NIR laser treatment induced an up-regulation of maturation markers including MHC class-II, CD40, and CD86 compared to controls injected i.d. with OVA only (Figure 4A–C, MHC II: *P* = 0.013; CD40: *P* = 0.010: CD86: *P*<0.037), but did not increase CD80 expression or OVA-positive DC populations above those of the controls (Figure 4D and 4E). To further determine the impact of NIR laser exposure on DC trafficking in the skin, we treated the skin of CD11c-YFP transgenic mice, in which DCs are intravitally labeled [35], [36], with a 1-minute exposure of the CW 1064 nm laser. We observed an over 2-fold increase in the concentration of DCs in both the epidermal and dermal areas of exposure, reaching a maximum 6 hours after treatment (Figure 5A and 5B, no laser control vs. laser treated in epidermis: 589±125 vs. 1,045±113/mm^2^, *P* = 0.038; dermis: 195±36 vs. 415±62/mm^2^, *P* = 0.040), and returning to the baseline level of non-treated skin by 24 hours. These data suggest that key mechanistic elements for the NIR laser include migrational and functional changes of DCs in skin and draining lymph node.

### NIR laser adjuvant results in the transient expression of a defined set of chemokines

After establishing the beneficial effect of NIR laser upon DCs, we sought to identify the mechanisms contributing to the migration and activation of DCs by the NIR laser. Skin cells function as sentinels for damage or pathogen invasion by releasing pro-inflammatory cytokines to recruit and condition APCs including DCs [45]. We therefore tested the effect of NIR laser upon chemokine production and signaling. We measured the expression of 160 genes related to inflammatory cytokines, their receptors, and inflammasomes using qPCR, 6 hours after the 1-minute CW 1064 nm laser treatment, Gene expression for a selective set of cytokines, including *Ccl2* and *Ccl20*, increased significantly (Figure 5C and Table S1). CCL2 and CCL20 protein expression has been shown to be involved in DC migration and recruitment [48]. 6 hours after the laser treatment, CCL2 was expressed in the epidermal and dermal regions, possibly in keratinocytes, fibroblasts and mast cells (Figure 5D, top row). CCL20 was expressed sporadically in the dermis, possibly in mast cells (Figure 5D, middle row). The expression of both CCL2 and CCL20 declined at 24 hours, matching the timing of DC localization to the laser-treated skin. Taken together, these data indicate that the NIR laser adjuvant stimulates the expression of a defined set of cytokines and chemokines which collectively could induce functional and migrational changes in DCs in the skin.

### The NIR laser adjuvant enhances humoral immunity without inducing an IgE response

**Figure 6:**
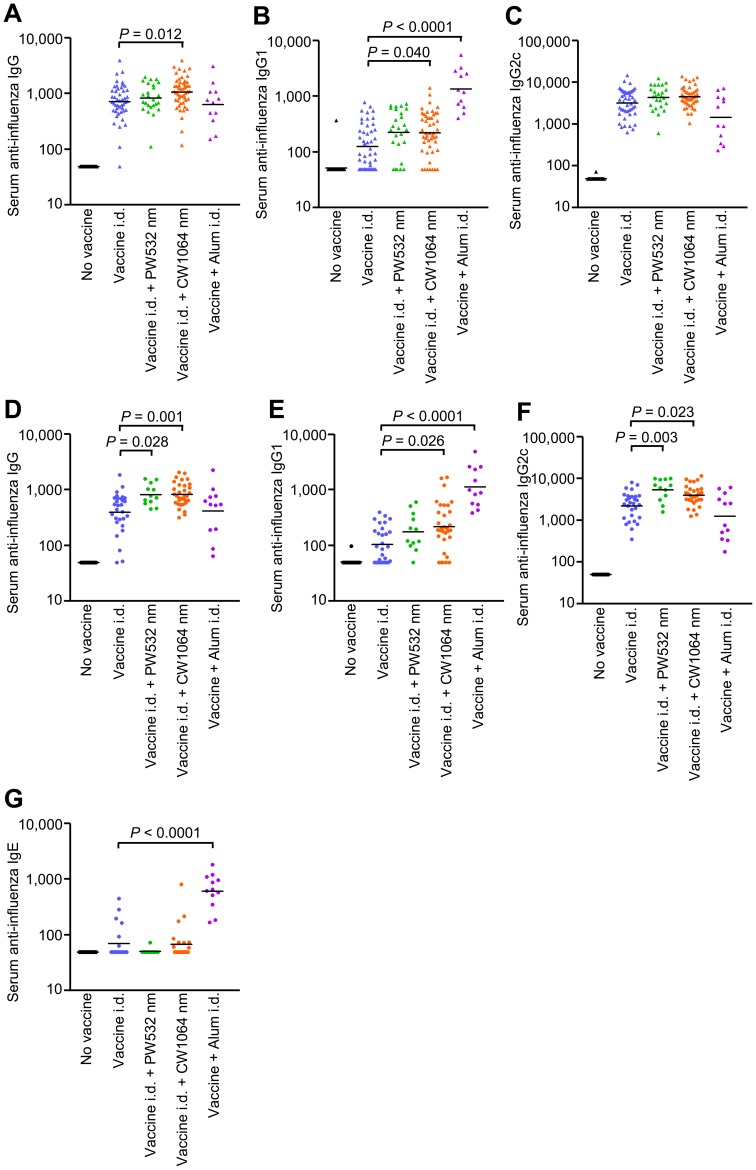
Effect of the laser adjuvant on humoral anti-influenza immune responses. A–G, Influenza-specific IgG subclass titers (A–C) in pre-challenge serum (4 weeks after vaccination) and (D–G) post-challenge (4 days after challenge). Mice were vaccinated with 1 µg of inactivated influenza virus (A/PR/8/34) with or without laser illumination or the licensed chemical adjuvant (alum) and challenged intranasally with live homologous virus 4 weeks after vaccination. Titer of influenza-specific serum IgG subclass was determined by ELISA. Plates were coated with inactivated influenza virus. (A and D) IgG, (B and E) IgG1, (C and F) IgG2c, and (G) IgE titers. Experimental and control groups: (A–C) *n* = 38, 47, 25, 50, 12 (D–F) *n* = 25, 29, 12, 32, 12 (G) *n* = 24, 21, 13, 24, 12 for no vaccine, vaccine i.d., vaccine i.d. + PW 532 nm, vaccine i.d. + CW 1064 nm, and vaccine + Alum i.d. vaccine groups, respectively.

We next examined the adjuvant effect of visible and NIR lasers in a murine influenza vaccination and lethal challenge model and compared them with alum. Mice received a single laser dose and were injected i.d. with whole inactivated influenza virus A/PR/8/34. We also used alum-adjuvanted vaccine to examine whether the laser adjuvant can induce responses comparable to a licensed chemical adjuvant, as i.d. injection of alum has been used to increase the efficacy of vaccines both in mice [39]–[42] and humans [43], [44]. The CW 1064 nm laser significantly augmented pre-challenge IgG and IgG1 titers compared to the non-adjuvanted group (Figure 6A–C, IgG: 1064 nm vs. controls: *P* = 0.012, IgG1: 1064 nm vs. controls: *P* = 0.040). Alum also produced elevations in IgG1 titers that were greater than non-adjuvanted controls (*P*<0.0001). The IgG2c responses were similar among all test groups. Post-challenge, the CW 1064 nm laser significantly augmented anti-influenza IgG, IgG1 and IgG2c titers compared to the non-adjuvanted group (Figure 6D–F, IgG: *P* = 0.001; IgG1: *P* = 0.026; IgG2c: *P* = 0.023). In comparison, the PW 532 nm laser augmented only IgG and IgG2c titers (IgG: *P* = 0.028; IgG2c: *P* = 0.003), and alum increased only IgG1 titers (*P*<0.001). The finding that alum elicited an IgG1-biased response is consistent with published literature showing that alum induces a profoundly polarized T helper type 2 (T_H_2) immune response with consequently elevated IgE production and hypersensitivity in mice [3], [10], [15], a finding replicated in this study (Figure 6G, *P*<0.0001 compared to the non-adjuvanted group). In contrast, the laser adjuvants did not increase IgE responses to the vaccine (Figure 6G). Influenza-specific IgA in the lung homogenate was not detected in any experimental group (data not shown), suggesting mucosal IgA does not contribute to protection in this model. These data indicate that NIR laser treatment produces a mixed T_H_1-T_H_2 immune response to influenza vaccination without enhancing an IgE response.

### NIR laser adjuvant induces a balanced systemic T_H_1-T_H_2 cell-mediated immune response

**Figure 7:**
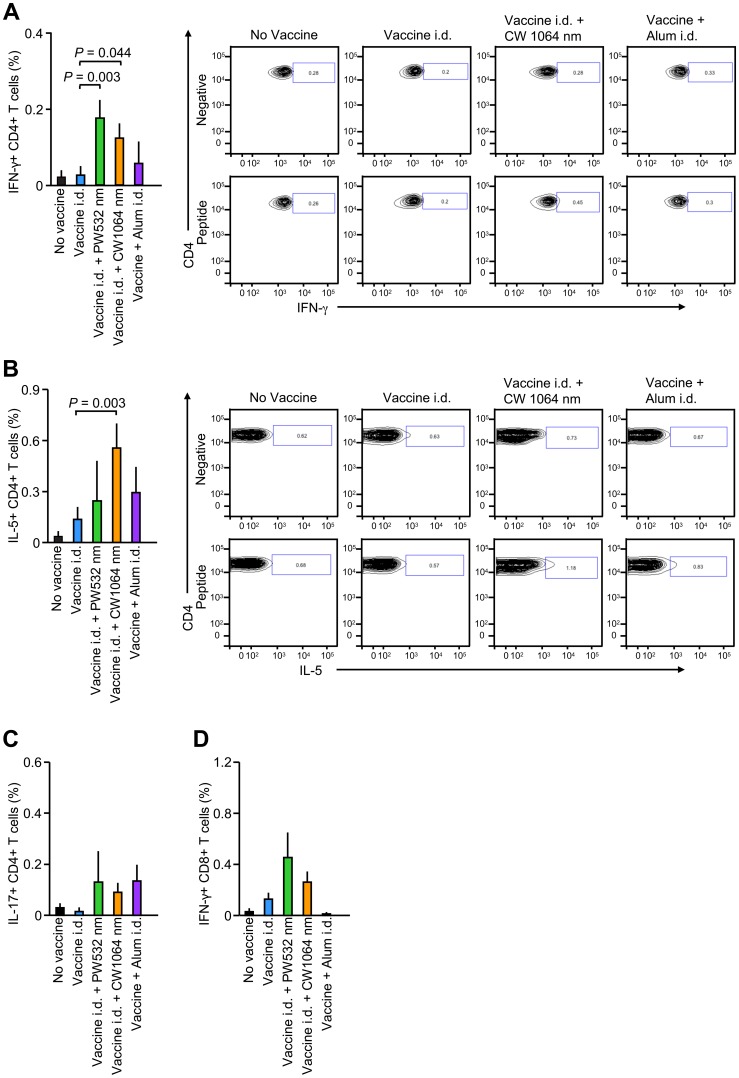
Effect of the laser adjuvant on cell-mediated anti-influenza immune responses. Systemic CD4+ helper T-cell responses were measured 4 days after challenge by re-stimulating 2×10^6^ splenocytes with a nucleoprotein (NP) major histocompatibility class-II complex (MHC) or class-I influenza-specific peptide. Percentages of (A) CD4+IFN-γ+ (B) CD4+IL-5+ (C) CD4+IL-17+ and (D) CD8+IFN-γ+ T cells are shown. Also shown are representative FACS plots. Error bars show means ± s.e.m. Experimental and control groups: (A–D) *n* = 16, 18, 13, 20, 10 for no vaccine, vaccine i.d., vaccine i.d. + PW 532 nm, vaccine i.d. + CW 1064 nm, and vaccine + Alum i.d. vaccine groups, respectively.

Previous studies suggest that effector CD4+ and CD8+ T cell-mediated immune responses contribute to protection from influenza [49], [50]. To determine whether the NIR laser elicits these cell-mediated immune responses, we re-stimulated splenocytes from influenza-challenged mice *ex vivo* with influenza peptides and then assessed the expression of cytokines in T-cell subpopulations. Influenza-specific CD4+IFN-γ+ T-cell subpopulations induced by a major histocompatibility complex (MHC) class-II peptide were significantly increased in the 1064 and 532 nm laser-treated groups compared to non-adjuvanted controls (Figure 7A, CW 1064 nm laser: *P* = 0.044; PW 532 nm laser: *P* = 0.003). Only the CW 1064 nm laser also significantly increased influenza-specific CD4+IL-5+ T-cell subpopulations (Figure 7B, *P*<0.003). No test groups showed significantly increased responses of influenza-specific CD4+IL-17+ T-cell subpopulations to MHC class-II peptide, or CD8+IFN-γ+ T-cells to MHC class-I peptide, compared to the non-adjuvanted controls (Figure 7C and 7D). These data show the unique ability of the non-tissue damaging NIR laser to induce a systemic T_H_1-T_H_2 immune response to an inactivated influenza vaccine.

### NIR laser adjuvant confers equivalent protective immunity to alum

**Figure 8:**
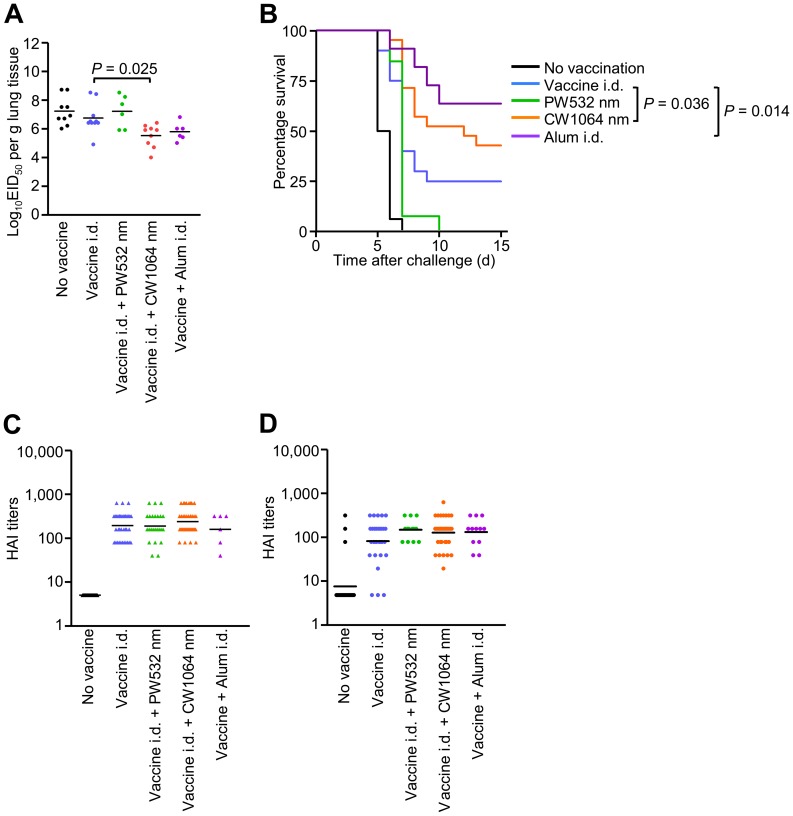
Effect of the laser adjuvant on protective immunity. A, EID_50_ was determined by serial titration of lung homogenate in eggs at 4 days after challenge. EID_50_, the 50% egg infectious dose. B, Kaplan-Meier survival plots of influenza-vaccinated mice for 15 days following lethal challenge; Gehan-Breslow-Wilcoxon test. C, HAI titers (C) in pre-challenge (4 weeks after vaccination) and (D) post-challenge (4 days after challenge) serum. Experimental and control groups: (A) *n* = 9, 10, 6, 9, 6 (B) *n* = 16, 20, 13, 21, 11 (C) *n* = 31, 38, 24, 41, 6 (D) *n* = 23, 31, 13, 34, 12 for no vaccine, vaccine i.d., vaccine i.d. + PW 532 nm, vaccine i.d. + CW 1064 nm, and vaccine + Alum i.d. vaccine groups, respectively.

After determining the quality of the laser-induced immune response, we sought to test the ability of this strategy to generate a protective response against lethal influenza virus challenge. Mice were vacciniated and allowed to rest for four weeks. Subsequently, naïve and vaccinated mice were challenged intra-nasally with homologous live influenza virus and monitored for survival time. In the lethal challenge model, the CW 1064 nm laser-treated group showed a marked decrease in lung viral titers by a factor of 10^1.9^ compared to the non-adjuvanted group at 4 days after challenge (Figure 8A, *P* = 0.025), while the PW 532 nm laser-treated group failed to show any significant impact. In addition, the single one minute duration CW 1064 nm laser treatment consistently conferred better protective immunity while the PW 532 nm laser treatment did not, as determined by survival time after viral challenge (Figure 8B, *P* = 0.036). Body weight loss upon viral challenge in CW 1064 nm laser-treated group was smaller than in PW 532 nm laser-treated group (Figure S2). There was no significant difference in protection and body weight loss between CW 1064 nm laser-treated and alum-adjuvanted groups. Consistent with protection and antibody levels, clinically relevant HAI titer levels in the CW 1064 nm laser group were higher than in the non-adjuvanted, 532 nm laser-treated and alum-adjuvanted groups in pre-challenge serum (Figure 8C), and higher than the non-adjuvanted group and comparable to those in the alum-adjuvanted group in post-challenge serum (Figure 8D). These data support the view that NIR laser induces protective immune responses to an inactivated influenza vaccine.

## Discussion

We have shown, for the first time, that non-tissue damaging NIR laser light given in short exposures to small areas of the skin, without the use of any additional agents, increases a broad spectrum of immune responses to influenza antigen. This occurs at a magnitude comparable to a licensed adjuvant, and results in improved survival in a lethal challenge murine model. NIR lasers have been used at high wattages in the field of medicine for decades [38]. Millions of people have been treated with 1064 nm lasers for tattoo and hair removal, skin tightening and regeneration at much higher powers than described in this study. The irradiances used in the study (in both mice and humans) are 10-fold less than those used for FDA-approved and safe cosmetic 1064 nm laser applications. The immune adjuvant settings would therefore be expected to be safer than NIR laser settings for cosmetic tissue destructive hair follicle and tattoo removal. To our knowledge, there are only two published reports of adverse effects related to these applications including a possible allergic response after Nd:YAG treatment. However, these were delayed type hypersensitivity reactions against the tattoo ink in the skin [51], [52]. Therefore, the finding that the exposure of a small area of skin to low-wattage NIR laser light is both tolerable and safe in humans could support rapid approval of this approach by the FDA.

The use of a low-power NIR laser with CW output has several clear advantages over visible lasers previously explored as adjuvanting devices [25], [26], as well as currently approved chemical vaccine adjuvants. Since water is the predominant chromophore for the NIR laser, light absorption is not significantly altered across different skin phototypes [53]. Further, the laser is external to a vaccine, avoiding stability issues that complicate conventional vaccine-chemical adjuvant combinations. Low-wattage CW 1064 nm NIR lasers are a mature, safe, compact and relatively simple technology, making it possible to economically produce a portable (handheld) low cost device that, at sufficient vaccination volumes, could offer a feasible alternative to chemical adjuvants. In addition, the NIR laser adjuvant could be readily translated into a safe, clinically applicable, approach as NIR laser devices already approved by the FDA- and European Medicines Agency- (EMEA-) could perform this function. All of these factors would reduce the logistical challenges of mass vaccination campaigns of underserved or outlying populations. Finally, the NIR laser greatly reduces potential adjuvant reactogenicity and toxicity, as it neither induces a prolonged inflammatory cytokine response nor promotes allergenicity, all while not persisting in exposed tissue. These features of a NIR laser adjuvant stand in contrast from well recognized drawbacks of chemical adjuvants [54].

We demonstrated that a single one-minute application of NIR 1064 nm laser alone to the skin, in conjunction with an influenza vaccine, induced a robust T_H_1-T_H_2 balanced and potentially protective immune response [55]–[58]. This contrasts with a nanosecond PW, visible 532 nm laser which induced a T_H_1-skewed response with little impact on the IgG1 response. Furthermore, the NIR laser, given in conjunction with i.d. influenza vaccine, confers protective immunity that assists in viral clearance from the lung and abrogates the need for a chemical adjuvant. In contrast, the visible range 532 nm laser exhibited minimal capacity to clear the virus and failed to confer protection in a lethal challenge model. IgA on the mucosal surface and a local T_H_1 response play a key role in controlling infections [59]–[62]. However, IgA was not detected in lungs and a T_H_1 response was increased in both CW 1064 and PW 532 nm laser-treated groups in this model. This suggests that a T_H_2 response is essential in conferring protection by the laser adjuvant in the context of i.d. vaccination using inactivated vaccine as evidenced by the critical role of IgG1 subclass in protection [56], [63]. Comparable protection with a T_H_2 response could be obtained by use of licensed or candidate adjuvants including alum, but these chemical or biological adjuvants are generally too reactogenic when delivered by the i.d. route [19], [24]. Our data support the view that the NIR laser adjuvant has the ability to activate immune responses in the dermis and epidermis, and therefore has the potential to replace problematic chemical and biological adjuvants.

We show that the mechanisms of action of the NIR laser adjuvant involve the transient expression of a limited set of cytokines and chemokines, including CCL2 and CCL20, which are known to direct recruitment of DCs to laser-illuminated skin and regulate adaptive immune responses [45], [48]. Key mechanistic elements identified so far include migrational and functional changes in DCs in both skin and draining lymph nodes. Our dataset supports this mechanistic action and demonstrates that the effect on DCs is evident within 6 hours post laser application, and is associated with augmentation of an adaptive immune response. Moreover, these immune stimulatory effects are completed within 24 hours after exposure without a residual effect beyond this time that would be generated by retained chemical or biological adjuvant material that could stimulate additional adverse effects. Finally we show that a thermal mechanism, which is known to enhance immune responses as a result of tissue damage, does not mediate the efficacy of the NIR laser adjuvant.

The magnitude of a CD4+ T cell response has been shown to be proportional to the number of DCs as well as their quality when they reach the lymph node [64]. Although the NIR laser adjuvant enhances adaptive immune responses, it did not induce a detectable increase of DCs in skin-draining lymph nodes. The migration of mature DCs to the draining lymph nodes is regulated at the multiple steps by expression of chemokines and chemokine receptors which play a determinant role in the trafficking of DCs to lymph nodes through afferent lymphatic vessels [64], [65]. As shown in this study, the tissue response to the laser illumination resulting in expression of chemokines tapers down within 24 hours, which would be long enough to initiate DC migration and maturation *in situ* but may not provide DCs with an additional guidance cue to traffic into lymph nodes.

Both government and non-governmental organizations have established as long-term objectives the reduction or elimination of chemical adjuvants when possible, as well as the development of needleless vaccinations. The NIR laser vaccine adjuvant technology, which could be readily incorporated into practice as a low-cost and easy-to-use handheld device, opens a new pathway towards achieving the long-term objectives in clinical practice. When combined with skin-based and needle-free technologies such as microneedles and transcutaneous immunization (TCI) patches [18], [19], [66], [67], use of the NIR laser adjuvant could eliminate the need for any type of hypodermic needle.

In summary, the NIR laser-based adjuvant represents a novel technology that could offer a feasible alternative for chemical and biological adjuvants in vaccines while facilitating the use of intradermal needleless vaccination in the future.

## Supporting Information

Figure S1 **Non-tissue damaging parameters of the laser adjuvants.** Mice received continuous wave (CW) or nanosecond-pulsed (PW) 1064 nm or PW 532nm laser illumination on four areas of shaved and depilated back skin for up to three min. Surface skin temperature was monitored with an infrared thermometer. A–B, Images of the back of mice for visual inspection at 0 and 24 h after laser illumination. There was no visible damage detected when the irradiance was below 1.0 W/cm^2^ for the PW 532 nm laser or 5.0 W/cm^2^ for the PW 1064 nm lasers, as evidenced by erythema, tissue edema, or bruising. A–B, *n* = 1–4 (4–16 exposures in total) for each group. Representative images for each group are presented.
(TIF)

Figure S2 **Effect of laser illumination on body weight following viral challenge.** Mice were vaccinated intradermally with 1 µg inactivated influenza virus (A/PR/8/34) with or without laser illumination, or alum-adjuvant. 28 days later, the mice were intranasally challenged with homotypic virus. Body weights were monitored daily for 15 days. Mean body weight ± s.e.m. of each experimental group was determined at each time point. *n* = 16, 20, 13, 21, 11 for no vaccine, vaccine i.d., vaccine i.d. + PW 532 nm, vaccine i.d. + CW 1064 nm, and vaccine + Alum i.d. vaccine groups.
(TIF)

Table S1 **Effect of the NIR laser illumination on the cytokine and chemokine expression in the skin.** 6 hours after the 1-minute CW 1064 nm laser treatment, the laser-treated skin sections were excised and total RNA was extracted. We measured the expression of 160 genes related to inflammatory cytokines, their receptors and inflammasomes using a RT^2^ Profiler™ PCR Array System. The fold change in mRNA expression over sham-treated controls were normalized against housekeeping genes and calculated following the 2^–ΔΔCT^ method per the manufacturer's instructions.
(DOC)
