# Supplementary figures and images for: Near-Infrared Laser Adjuvant for Influenza Vaccine
Source: PLoS One. 2013 Dec 11;8(12):e82899. doi: 10.1371/journal.pone.0082899 (PMC3859633; doi:10.1371/journal.pone.0082899)

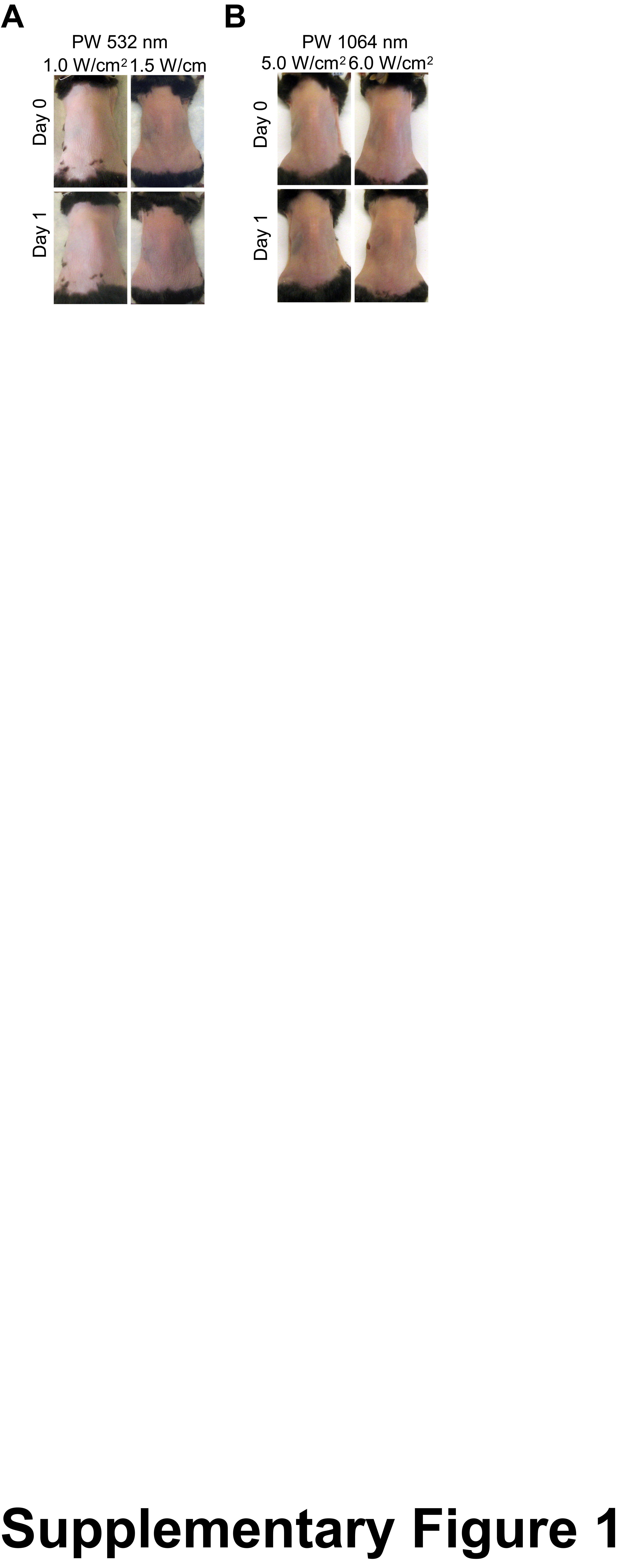

Supplement: Figure S1 — Non-tissue damaging parameters of the laser adjuvants. Mice received continuous wave (CW) or nanosecond-pulsed (PW) 1064 nm or PW 532nm laser illumination on four areas of shaved and depilated back skin for up to three min. Surface skin temperature was monitored with an infrared thermometer. A–B, Images of the back of mice for visual inspection at 0 and 24 h after laser illumination. There was no visible damage detected when the irradiance was below 1.0 W/cm2 for the PW 532 nm laser or 5.0 W/cm2 for the PW 1064 nm lasers, as evidenced by erythema, tissue edema, or bruising. A–B, n = 1–4 (4–16 exposures in total) for each group. Representative images for each group are presented. (TIF) [file pone.0082899.s001.tif]

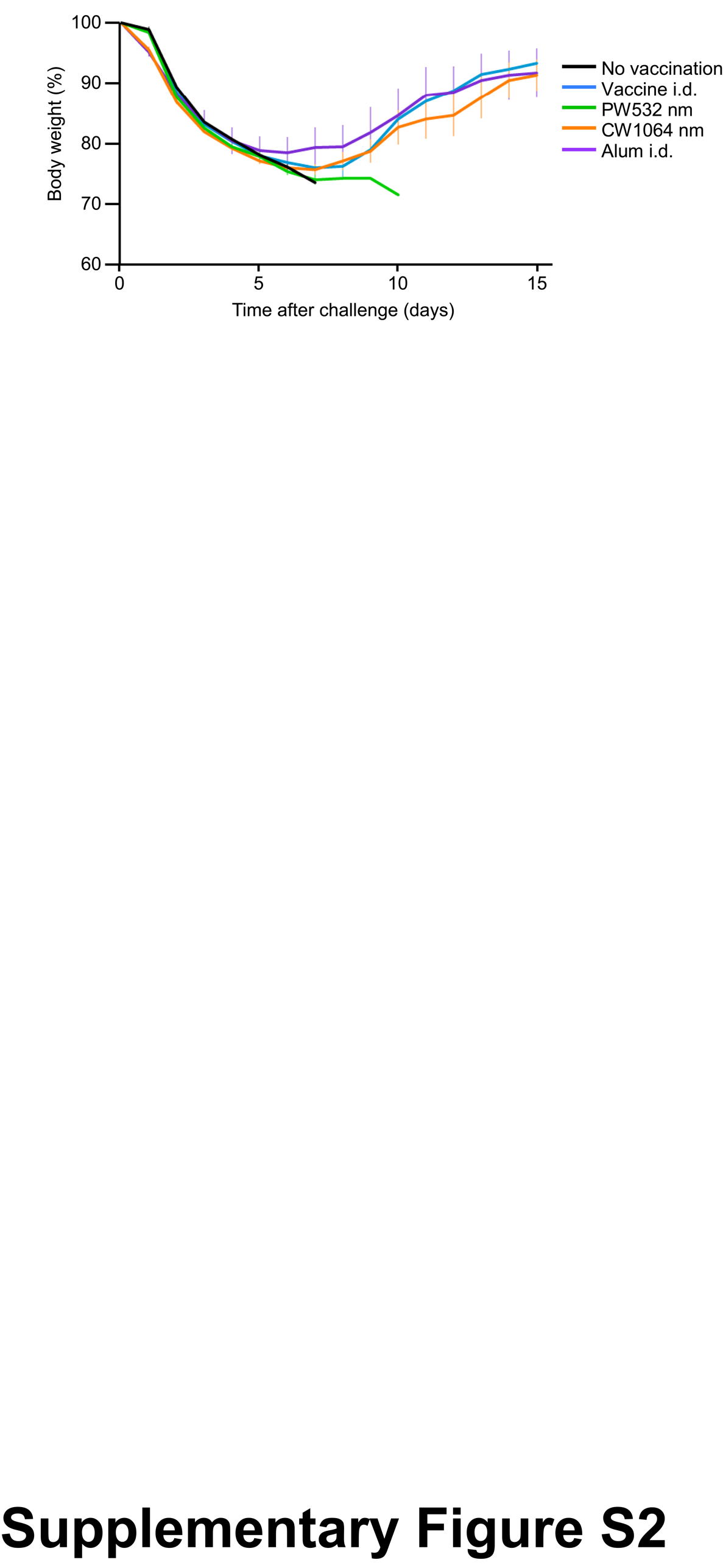

Supplement: Figure S2 — Effect of laser illumination on body weight following viral challenge. Mice were vaccinated intradermally with 1 µg inactivated influenza virus (A/PR/8/34) with or without laser illumination, or alum-adjuvant. 28 days later, the mice were intranasally challenged with homotypic virus. Body weights were monitored daily for 15 days. Mean body weight ± s.e.m. of each experimental group was determined at each time point. n = 16, 20, 13, 21, 11 for no vaccine, vaccine i.d., vaccine i.d. + PW 532 nm, vaccine i.d. + CW 1064 nm, and vaccine + Alum i.d. vaccine groups. (TIF) [file pone.0082899.s002.tif]
