# Supplementary material for: Near-Infrared Laser Adjuvant for Influenza Vaccine
Source: PLoS One. 2013 Dec 11;8(12):e82899. doi: 10.1371/journal.pone.0082899 (PMC3859633; doi:10.1371/journal.pone.0082899)
Supplement: Table S1 — Effect of the NIR laser illumination on the cytokine and chemokine expression in the skin. 6 hours after the 1-minute CW 1064 nm laser treatment, the laser-treated skin sections were excised and total RNA was extracted. We measured the expression of 160 genes related to inflammatory cytokines, their receptors and inflammasomes using a RT2 Profiler™ PCR Array System. The fold change in mRNA expression over sham-treated controls were normalized against housekeeping genes and calculated following the 2–ΔΔCT method per the manufacturer's instructions. (DOC) [file pone.0082899.s003.doc]

**Supplementary Table S1.** Effect of the NIR laser illumination on the cytokine and chemokine expression in the skin.

| Gene symbols | Fold increase | Gene symbols | Fold increase | Gene symbols | Fold increase | Gene symbols | Fold increase |
| --- | --- | --- | --- | --- | --- | --- | --- |
| *Ccl1* | 1.40 | *Ccr10* | 0.96 | *Cd40lg* | 0.73 | *Nfkbia* | 1.51 |
| *Ccl2* | *3.92 | *Il1a* | *3.28 | *Cflar* | 1.05 | *Nfkbib* | 1.16 |
| *Ccl3* | 1.51 | *Il1b* | 2.13 | *Chuk* | 1.20 | *Nlrc4* | 1.08 |
| *Ccl4* | 1.22 | *Il1f6* | 4.42 | *Ciita* | 0.79 | *Nlrc5* | 1.01 |
| *Ccl5* | 1.12 | *Il1f8* | 4.33 | *Ctsb* | 0.87 | *Nlrp1a* | 1.16 |
| *Ccl6* | *6.38 | *Il1r1* | 1.70 | *Crp* | 0.78 | *Nlrp3* | 1.53 |
| *Ccl7* | 2.04 | *Il1r2* | 0.65 | *C3* | 1.39 | *Nlrp4b* | 0.41 |
| *Ccl8* | 3.17 | *Il2rb* | 3.17 | *Fadd* | 1.03 | *Nlrp4e* | 0.41 |
| *Ccl9* | *1.85 | *Il2rg* | 1.04 | *Hsp90aa1* | 1.35 | *Nlrp5* | 0.41 |
| *Ccl11* | *3.31 | *Il3* | 0.72 | *Hsp90ab1* | 0.78 | *Nlrp6* | 0.65 |
| *Ccl12* | 2.36 | *Il4* | 0.81 | *Hsp90b1* | 1.09 | *Nlrp9b* | 0.41 |
| *Ccl17* | *6.35 | *Il5ra* | *0.37 | *Ifnb1* | *0.35 | *Nlrp12* | *0.34 |
| *Ccl19* | 1.16 | *Il6* | 5.43 | *Ifng* | 1.32 | *Nlrx1* | 1.46 |
| *Ccl20* | *1.78 | *Il6ra* | 0.99 | *Itgam* | 2.60 | *Nod2* | 1.46 |
| *Ccl22* | 2.32 | *Il6st* | 0.92 | *Itgb2* | 1.02 | *P2rx7* | 1.08 |
| *Ccl24* | 0.96 | *Il10* | 1.57 | *Ikbkb* | 1.30 | *Panx1* | 0.89 |
| *Ccl25* | 1.14 | *Il10ra* | 1.28 | *Ikbkg* | 1.02 | *Pea15a* | 0.58 |
| *Ccr1* | 2.37 | *Il10rb* | 1.04 | *Irak1* | 1.18 | *Pf4* | 2.84 |
| *Ccr2* | 4.00 | *Il11* | 1.05 | *Irf1* | 0.69 | *Pstpip1* | 0.72 |
| *Ccr3* | 3.20 | *Il12a* | 1.60 | *Irf2* | 0.86 | *Ptgs2* | *7.00 |
| *Ccr4* | 0.68 | *Il12b* | *0.44 | *Irf3* | 0.79 | *Pycard* | 1.67 |
| *Ccr5* | 2.65 | *Il13* | 0.59 | *Lta* | *0.47 | *Stk30* | 1.06 |
| *Ccr6* | 0.43 | *Il13ra1* | 1.43 | *Ltb* | 0.79 | *Rela* | 1.09 |
| *Ccr7* | *4.89 | *Il15* | 1.21 | *Map3k7* | 1.35 | *Ripk2* | 1.24 |
| *Ccr8* | 2.17 | *Il16* | 1.45 | *Mif* | 0.72 | *Spp1* | 1.26 |
| *Ccr9* | 2.21 | *Il17b* | 1.92 | *Tab1* | 0.91 | *Sugt1* | 1.19 |
| *Cxcl1* | 2.50 | *Il18* | 1.73 | *Tab2* | 0.94 | *Tirap* | 2.18 |
| *Cxcl3* | *0.28 | *Il33* | 2.06 | *Mapk1* | 1.04 | *Tnfrsf1a* | *2.31 |
| *Cxcl5* | 0.43 | *Il20* | 0.88 | *Mapk11* | 0.96 | *Tnfrsf1b* | 2.68 |
| *Cxcl9* | 2.52 | *Aimp1* | 0.98 | *Mapk12* | 0.63 | *Tollip* | 0.81 |
| *Cxcl10* | 1.88 | *Aim2* | 0.37 | *Mapk13* | 0.88 | *Tgfb1* | 1.91 |
| *Cxcl11* | 0.75 | *Abcf1* | 0.64 | *Mapk3* | 0.79 | *Tnf* | 1.41 |
| *Cxcl12* | 1.46 | *Bcl2* | 1.02 | *Mapk8* | 1.40 | *Tnfsf11* | 2.37 |
| *Cxcl13* | 5.45 | *Bcl6* | 1.66 | *Mapk9* | 0.96 | *Tnfsf14* | 1.00 |
| *Cxcl15* | 0.72 | *Bcl2l1* | 0.59 | *Mefv* | 1.37 | *Tnfsf4* | 0.97 |
| *Cx3cl1* | 0.42 | *Birc2* | 1.23 | *Myd88* | 1.01 | *Traf6* | 1.16 |
| *Cxcr2* | 4.11 | *Birc3* | 1.23 | *Naip1* | 0.52 | *Txnip* | 0.86 |
| *Cxcr3* | 1.09 | *Card6* | 0.86 | *Naip5* | 1.11 | *Xcr1* | 1.08 |
| *Cxcr5* | *0.23 | *Casp1* | 0.78 | *Nfkb1* | 1.03 | *Xiap* | 1.05 |

Values are fold-increases in mRNA expression compared to non-laser treated controls.

**P* < 0.05 as compared to non-laser treated controls.
